# Endotracheal Intubation Without Neuromuscular Blocking Agents: Is It a Good and Safe Option?

**DOI:** 10.5812/aapm.3805

**Published:** 2012-04-01

**Authors:** Mert Akan, Sermin Oztekin

**Affiliations:** 1 Department of Anesthesiology and Reanimation, Faculty of Medicine, Dokuz Eylul University, Izmir, Turkey

**Keywords:** Intubation, Intratracheal, Neuromuscular Blocking Agents

*Dear Editor,*

We read with great interest the article, “Use of Remifentanil and Alfentanil in Endotracheal Intubation: A Comparative Study,” by Imani *et al*. (1). The authors demonstrated that remifentanil in combination with propofol provides excellent conditions for endotracheal intubation in young, healthy patients to avoid the use of muscle relaxants. However, there are some points about the article’s focus and results that we would like to discuss in this commentary. Propofol in combination with an opioid has been used by many investigators to facilitate neuromuscular blocking agent-free intubation. In most of these studies, at doses of remifentanil 2 µg/kg and above, blood pressure and heart rate were significantly reduced compared with baseline levels; this anesthetic technique with high doses of remifentanil caused severe cardiac depression in adults (2-4). In this study, the authors used a very high dose of remifentanil (5µg/kg) but did not give any data about the hemodynamic variables. In the literature, there are still patients with unacceptable responses to intubation even after high doses of remifentanil. Klemola *et al*. showed that intubation was impossible in 25% and 5% of adult patients who received remifentanil 3 and 4 µg/kg, respectively (5). Although Imani *et al*. claimed that intubating conditions were quite suitable in most of the patients in this article, we noticed that there were two patients in the alfentanil group with a vocal cords’ patency score of 4 and 14 patients with a vocal cords’ patency score of 3 (in Table 3). We do not see how these patients had clinically acceptable intubating conditions, and we wonder if there were any patients who could not be intubated? Also, the authors did not provide any data about the adverse events related with laryngeal morbidity. Mencke *et al*. reported that the quality of tracheal intubation contributed to laryngeal morbidity, and excellent conditions were less frequently associated with postoperative hoarseness and vocal cord sequelae. In addition, they concluded that vocal cord sequelae occurred more frequently in patients who were intubated without muscle relaxants and that the addition of nondepolarizing muscle relaxants to a propofol–fentanyl induction regimen improved the quality of tracheal intubation and decreased postoperative hoarseness (6). In another study, Holzki *et al*. demonstrated that stridor was not a main outcome measure for assessing airway injury after extubation (7). Stridor might develop weeks or months after airway injury, and only endoscopy can clearly detect all airway injuries. Further, in a Danish report, Lundstrom *et al*. concluded that avoiding neuromuscular blocking agents might increase the risk of difficult tracheal intubation, which is often related with minor airway injuries (8). Therefore, anesthesiologists should aim for excellent intubating conditions in all cases to avoid causing laryngeal morbidity. Finally, the article concentrates on the technique to intubate the trachea without muscle relaxation. As we mentioned before, the literature describes some techniques to intubate the trachea without the use of neuromuscular blocking agents under general anesthesia. However; the question remains as to whether it is a safe and good option. Combes *et al*. compared two induction regimens using or not using a muscle relaxant and concluded that the use of a muscle relaxant for tracheal intubation diminishes the incidence of adverse postoperative upper airway symptoms, results in better tracheal intubation conditions, and reduces the rate of adverse hemodynamic events (9). In short procedures in which endotracheal intubation is mandatory but muscle relaxation for surgery is not required, such as certain Ear Nose Throat (ENT) and neurosurgical procedures, reduced doses of neuromuscular blocking agents can be used to facilitate the endotracheal intubation. Oztekin *et al*. suggested the use of a reduced dose of rocuronium (0.3 mg/kg) with propofol (2.5 mg/kg) and remifentanil infusion (0.5µg/kg/min) to obtain satisfactory intubating conditions without any cardiovascular instability in pediatric day-case surgery (10). Furthermore, sugammadex, a new agent that allows prompt reversal of both rocuronium and vecuronium, should be kept in my mind for very short procedures as well as certain neuromuscular disorders in which reduced doses of rocuronium can be used to facilitate endotracheal intubation. Succinylcholine is also an alternative depolarizing neuromuscular blocking agent for short procedures and rapid sequence induction (crush intubation). However, succinylcholine is contraindicated in certain conditions (e.g., choline-esterase enzyme deficiency, recent spinal cord injury, burns, hyperkalemia, susceptibility to malignant hyperthermia). In rapid sequence induction, high doses of rocuronium (1 to 1.2 mg/kg) can be used as an alternative to succinylcholine, but if the procedure is very short, sugammadex can help to reverse the neuromuscular blockage. Nevertheless, in practice, there may be some cases in which both depolarizing and nondepolarizing muscle relaxants are contraindicated and sugammadex is not available; in such cases avoidance of neuromuscular blocking agents for intubation can be a valid or even unavoidable technique in the anesthetist’s armamentarium. Therefore, the authors’ attempt in this research area is commendable.
